# Immune Microenvironment and Response in Prostate Cancer Using Large Population Cohorts

**DOI:** 10.3389/fimmu.2021.686809

**Published:** 2021-10-28

**Authors:** Xiaohan Ren, Xinglin Chen, Xu Zhang, Silin Jiang, Tongtong Zhang, Guangyao Li, Zhongwen Lu, Dong Zhang, Shangqian Wang, Chao Qin

**Affiliations:** ^1^ The State Key Lab of Reproductive Medicine, the First Affiliated Hospital of Nanjing Medical University, Nanjing, China; ^2^ Department of Urology, the First Affiliated Hospital of Nanjing Medical University, Nanjing, China

**Keywords:** prostate cancer, immune, prognosis, immunotherapy, response rate

## Abstract

Immune microenvironment of prostate cancer (PCa) is implicated in disease progression. However, previous studies have not fully explored PCa immune microenvironment. This study used ssGSEA algorithm to explore expression levels of 53 immune terms in a combined PCa cohort (eight cohorts; 1,597 samples). The top 10 immune terms were selected based on the random forest analysis and used for immune-related risk score (IRS) calculation. Furthermore, we explored differences in clinical and genomic features between high and low IRS groups. An IRS signature based on the 10 immune terms showed high prediction potential for PCa prognosis. Patients in the high IRS group showed significantly higher percentage of immunotherapy response factors, implying that IRS is effective in predicting immunotherapy response rate. Furthermore, consensus clustering was performed to separate the population into three IRSclusters with different clinical outcomes. Patients in IRScluster3 showed the worst prognosis and highest immunotherapy response rate. On the other hand, patients in IRScluster2 showed better prognosis and low immunotherapy response rate. In addition, *VGLL3*, *ANPEP*, *CD38*, *CCK*, *DPYS*, *CST2*, *COMP*, *CRISP3*, *NKAIN1*, and *F5* genes were differentially expressed in the three IRSclusters. Furthermore, CMap analysis showed that five compounds targeted IRS signature, thioridazine, trifluoperazine, 0175029-0000, trichostatin A, and fluphenazine. In summary, immune characteristics of PCa tumor microenvironment was explored and an IRS signature was constructed based on 10 immune terms. Analysis showed that this signature is a useful tool for prognosis and prediction of immunotherapy response rate of PCa.

## Introduction

Prostate cancer (PCa) is the most common urological malignant tumor in men and the second leading cause of cancer-related death in men in the Western world (1). Currently, radical prostatectomy is the conventional treatment approach for localized PCa. However, 25%–30% PCa patients who undergo radical prostatectomy progress to advanced disease stage associated with high recurrence and poor prognosis within 10 years (2). In addition, most metastatic PCa do not undergo full remission and are associated with severe symptoms as a result of osseous metastases, despite long-term survival (3). Androgen deprivation therapy (ADT), such as enzalutamide and abiraterone, is the conventional approach for treatment of advanced and metastasized PCa. Although most patients initially show high response rate to hormone therapy, they invariably evolve to castrate-resistant prostate cancer (CRPC) after several years. CRPC is the lethal form of prostate cancer and is associated with poor prognosis (4). CRPC patients undergo chemoradiotherapy such as cyclophosphamide and methotrexate, yet the approach is associated with limited efficacy and severe side effects (5). The limitations for current therapy approaches call for the need to explore potential mechanism of PCa progression and novel targets to improve therapeutic intervention.

In the tumor microenvironment (TME), crosstalk of different cells like tumor cells, immune cells, and stromal cells and other noncellular components play a role in tumor progression (6). Immune cells comprise many cell subsets differentiated from hematopoietic stem cells and play important role in TME and immune response. Heterogeneous immune cells maintain tissue homeostasis through immune regulation and killing of tumor cells mediated by cell interaction and cytokine signaling (7). Several studies have explored the role of various immune cells in tumor tissue on tumor initiation and progression (8). Immunohistochemistry staining of gastric and gastro-oesophageal junction adenocarcinomas, for example, showed an increase in CD8+ T cell which was associated with poor prognosis. In addition, higher PD-L1 level was observed which implies an underlying immune resistance mechanism (9). Furthermore, Zappasodi and their colleagues reported that dysregulation of PD-1 and CTLA4 checkpoint receptors were both constitutively upregulated in Treg cells, implying that they have an immunosuppressive function (10). A previous study reports that follicular B cells in tertiary lymphoid structures improve survival of nonsmall cell lung cancer patients (11). Recently, application of immunotherapy to manipulate the patient’s immune system to fight tumor cells is a promising approach for cancer treatment. For instance, Sabado et al. explored the role of dendritic cells (DC) in tumor therapy and developed DC vaccines based on the results (12). DCs were mostly involved in antigen presentation and mainly derived from *ex vivo*-generated monocyte in the clinical trial. The DCs were infused into the body through different ways, numbers, and times to induce an enhanced and persistent immune response. In addition, the potential of other immune cells like NK cells and T cells in immunotherapy development has been explored (13, 14). The role of immune microenvironment in PCa progression, and successes reported in tumor immune therapy, drives the need to identify immune characteristics and biomarkers associated with the survival of PCa patients. However, studies have not explored immune-infiltrating features in PCa tissue and their correlation with immune therapy response.

In this study, RNA-seq data and clinical information were collected from eight independent prostate adenocarcinoma (PRAD) cohorts with 1,597 samples. Furthermore, 53 immune terms were quantified using single-sample gene set enrichment analysis (ssGSEA) algorithm. The top 10 significant immune terms were used in the calculation of immune-related risk score (IRS) and construction of an IRS signature. The IRS signature effectively predicted prognosis and immunotherapy response rate of PCa patients. In addition, consensus clustering was performed resulting in three IRSclusters. These clusters showed diverse survival outcomes and response rates to immunotherapy. Furthermore, *VGLL3*, *ANPEP*, *CD38*, *CCK*, *DPYS*, *CST2*, *COMP*, *CRISP3*, *NKAIN1*, and *F5* genes in these three IRSclusters showed a regular cluster-specific expression pattern. Moreover, connectivity map (CMap) analysis was performed to identify potential compounds that target the IRS signature, which can be used for PCa treatment.

## Methods and Materials

### PCa Data Retrieval and Preprocessing

A comprehensive search was performed on public databases for available expression matrix data, and complete clinical annotations of PCa patients were matched. A total of eight PCa cohorts (The Cancer Genome Atlas (TCGA)-PRAD, German Cancer Research Center (DKFZ)-PRAD, GSE116918, GSE46602, GSE70768, GSE70769, Memorial Sloan Kettering Cancer Center (MSKCC)-PRAD, Stand up to Cancer Prostate Cancer Foundation (SU2C_PCF)-PRAD) were selected for further processing and analysis. TCGA-PRAD data were retrieved from TCGA-GDC database (https://portal.gdc.cancer.gov/). The screening criteria for the PCa cohort in the GEO dataset (https://www.ncbi.nlm.nih.gov/geo/) were as follows: (1) more than 45,000 probes in the platform annotation file to ensure sufficient gene symbol; (2) value of all probes in each sample greater than 0; (3) availability of detailed prognosis information for all patients; (4) publicly available gene expression profile; and (5) number of samples equal or greater than 50. Four GSE datasets (GSE46602, GSE70768, GSE70769, and GSE116918) met the criteria and were thus used for analysis. In addition to TCGA and GSE databases, other PCa expression profiles and clinical features (DKFZ-PRAD, MSKCC-PRAD, SU2C_PCF-PRAD) were retrieved from the cBioPortal webserver (https://www.cbioportal.org/). RNA sequence data were initially retrieved with FPKM value and then transformed into transcripts per kilobase million (TPM) type for higher comparability with microarray data (15, 16). Genes with a low abundance in most samples were removed, to ensure the RNA-sequence matrix is closer to the signal strength chip (17). Missing clinical data in our PCa cohort were retrieved by (1) trying to contact authors to get missing data and (2) carefully examining supplementary files for relevant literature. If multiple probes corresponded to the same gene symbol, the average value of these probes was calculated as the representative expression level of this gene. Normalization process resulted in a range between 0 and 25 of the expression value of eight cohorts for a better combination. Combat algorithm in the sva package was used to correct intra- and interbatch effect (18). IMvigor210 cohort, a urothelial carcinoma cohort treated with the anti‐PD‐L1 antibody atezolizumab, was used for prediction of patient response to immunotherapy. It was downloaded from a freely available, fully documented software and data package, under the Creative Commons 3.0 license that can be downloaded from http://research-pub.gene.com/IMvigor210CoreBiologies. ConsensusClusterPlus package was used to distinguish IRS subgroups of PRAD with resamplings set as 1,000 (https://figshare.com/articles/software/Clustering/16531407).

### Gene Set Enrichment Analysis and Gene Set Variation Analysis

Gene set enrichment analysis (GSEA) was performed using the “clusterProfiler” package in R software (version 3.6.1), which is a knowledge-based approach for interpreting genome-wide expression profiles. ssGSEA, a module in the gene set variation analysis (GSVA) package, was performed to quantify normalized enrichment score (NES) of 53 immune cells and immune response (19). The gene set used to quantify 53 immune terms has been uploaded in Figshare (https://figshare.com/articles/dataset/Immune_set/14286632). Gene set files (.gmt) of C1–C8 and Hallmark were all retrieved from MSigDB (20).

### Immune-Related Risk Score for the Combined PCa Cohort

TCGA-PRAD, GSE70768, and GSE70769 cohorts were selected as the training cohort due to their similar annotation platform (Illumina HumanHT-12 V4.0 expression beadchip), and other datasets were selected as the validation cohort (GSE46602, DKFZ-PRAD, GSE116698, MSKCC-PRAD, and SU2C-PRAD). Prognosis-related immune terms was first identified from 53 immune terms through univariate Cox analysis with the screening criteria *p*-value <0.05 of the training cohort. Supervised regression random forest algorithm in the R package “randomForestSRC” was used to conduct dimension reduction (ntree = 1,000). The top 10 significant genes were then selected for multivariate Cox regression analysis and IRS calculation using the following formula:


Immune risk score=β1μ1+β2μ2+β3μ3… …βNμN

where “*β*,” “*μ*,” and “*N*” represent the coefficient, NES value, and number of selected immune terms, respectively. The R packages SimDesign and tdROC were used to conduct logistic regression for the best cutoff value. The prognosis value was assessed through Kaplan-Meier survival curve and ROC curve.

### Genomics Features and TIDE Score

Tumor mutation burden (TMB) represents the number of base mutations per 1 Mb length. TMB was calculated using mutation data (.maf; C>T, C>G, C>A, T>G, T>C, T>A) retrieved from TCGA-PRAD database. Microsatellite instability (MSI) of PRAD patients was determined from previously sorted data induced by defects in the mismatch repair system (21). Expression profile of immune-related genes was directly extracted from the gene matrix. Copy number variation (CNV) segment files were downloaded from firehose (version.hg19; http://gdac.broadinstitute.org). GISTIC 2.0 was used to calculate the gistic score (https://cloud.genepattern.org). The two output files “focal_data_by_genes.txt” and “broad_data_by_genes.txt” were used to calculate the CNV burden in R software (Focal and Arm-level). One-class logistic regression was used to calculate the stemlike indices of each TCGA-PRAD patient following a procedure reported in a previous study (22). Proportions of estimate and immune cells in tumor tissue were quantified using the estimate package in R software. TIDE is an algorithm developed for modeling immune evasion and predicting immunotherapy response in tumor patients by integrating the characteristics of T-cell dysfunction and T-cell exclusion (23). The normalized expression profile was analyzed using the TIDe webserver (http://tide.dfci.harvard.edu/). Each patient was assigned a TIDE score where >0.2 was defined as no responder and <−0.2 was defined as responder.

### Potential Compounds Targeting the PRAD Immune Signature

CMap dataset is a collection of genome-wide transcriptional expression data from cultured human cells treated with small bioactive molecules that is available at the Broad Institute (24). The data and attern-matching algorithms provide information on underlying association between drugs, genes, and diseases through the transitory feature of common gene-expression changes. Differentially expressed genes (DEGs) were identified between patients in the top 50 and lowest 50 IRS groups. DEGs were then used for CMap analysis. The small bioactive molecules that were potential targets for PRAD immune signature were screened usings the following filtering criteria: (1) PC-3 cell line (prostate cancer cell lines); (2) a trial number ≥2; (3) an enrichment score <−0.6 and *p*-value <0.05; and (4) percent nonnull = 100.

### Statistical Analysis

All analyses were performed using R v3.6.1 and SPSS v23 software. All statistical tests were two sided, and *p*-value <0.05 was considered statistically significant. An independent *t*-test was used to compare continuous variables of normal distribution, and Wilcoxon rank-sum test was used to compare continuous variables with skewed distribution. Spearman analysis was performed to determine correlation coefficients. DEGs were identified with the limma package from the TPM data in the gene matrix (25). The R package ggplot2 was used to generate plots. Kaplan-Meier survival and ROC curve were used to determine survival of patients using timeROC package.

## Results

### Calculation of NES of 53 Immune Terms Based on the mRNA Expression Profile

**Figure 1:**
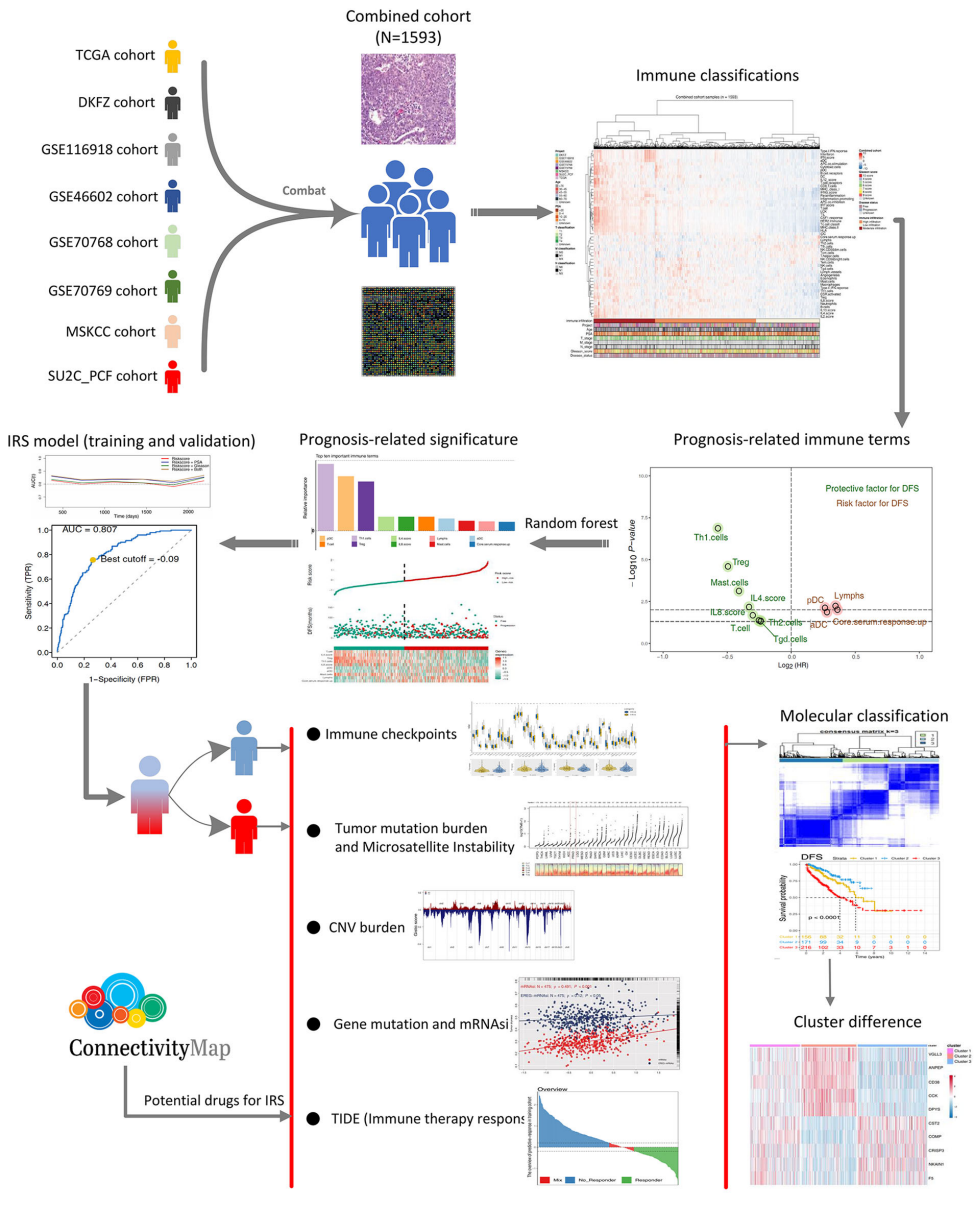
The whole flowchart of the analysis based on large PCa cohorts. Eight independent PCa cohorts were included for our analysis and further combined into a large population PCa cohort using sva package. Expression profile of all patients was quantified as 53 immune terms using ssGSEA package. Top 10 important immune terms identified from random forest algorithm were used for IRS caculation. Then, we found that high IRS patients showed poor clinical features, genomic character, and biological pathway associated with poor prognosis of PCa poor clinical outcomes. Meanwhile, it also could predict the immunotherapy response rate of PCa patients.

**Figure 2:**
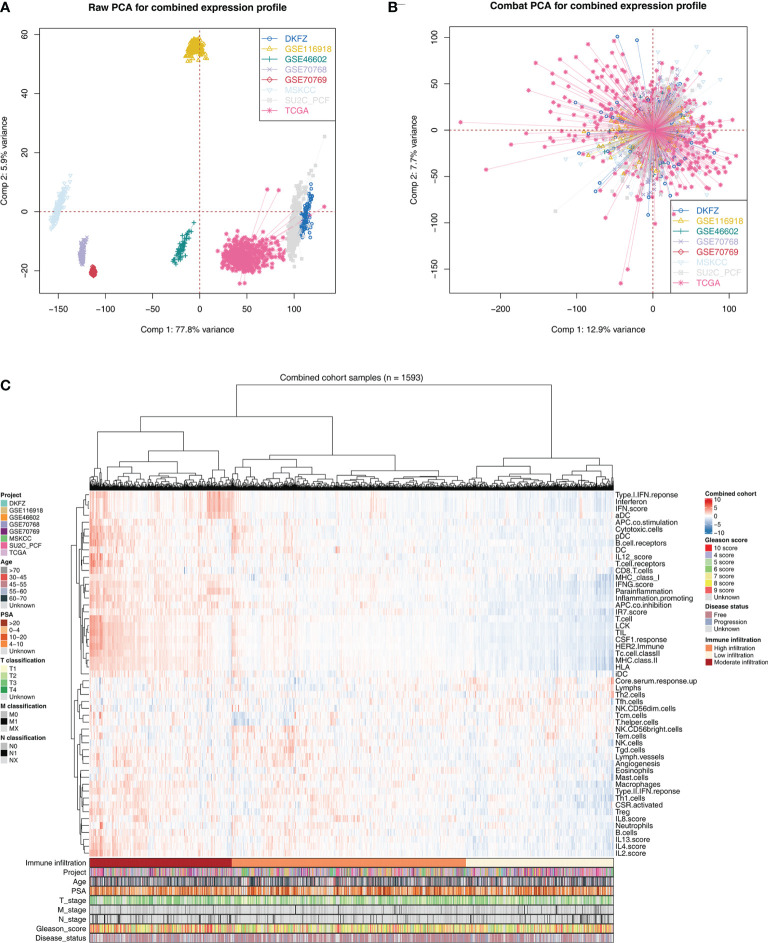
Combination of PCa cohort and quantification of immune terms. **(A)** Eight PCa cohort selected for our analysis have obvious batch difference. **(B)** The sva package used for PCa cohort combination greatly reduces the batch difference. **(C)** Expression profile of all patients was quantified as 53 immune terms using ssGSEA package. These immune terms were shown in the heatmap with their corresponding clinical features.

The flowchart of the whole analysis is shown in **Figure 1**. Following a comprehensive screening of cBioPortal, GEO, and TCGA public databases, eight PRAD cohorts met the study criteria and were included for analysis (**Table 1**). The eight cohorts showed a significant batch difference (**Figure 2A**). Sva package was used to remove batch effect for the eight PRAD cohorts and to generate a combined PRAD cohort with a large population (1,597 tumor samples) (**Figure 2B**). Expression profile of all patients was quantified as 53 immune terms using the ssGSEA package (**Figure 2C**).

**Table 1:** Detailed information of PCa cohort used for combination in the analysis.

Cohort	Platform	Number of input	Data source	Age	PSA (ng/ml)	Gleason
TCGA-PRAD	Illumina HumanHT-12 V4.0 expression beadchip	485	National Cancer Institute	60.97 ± 6.84	1.49 ± 15.01	7.60 ± 1.01
GSE70768	Illumina HumanHT-12 V4.0 expression beadchip (GPL10558)	199	Cancer Research UK Cambridge Institute	38.84 ± 30.61	11.34 ± 34.47	6.92 ± 1.32
GSE70769	Illumina HumanHT-12 V4.0 expression beadchip (GPL10558)	94	Cancer Research UK Cambridge Institute	NA	10.81 ± 13.13	6.81 ± 1.54
GSE46602	[HG-U133_Plus_2] Affymetrix Human Genome U133 Plus 2.0 Array (GPL570)	50	Department of Urology, Aarhus University Hospital	62.59 ± 6.27	18.15 ± 10.11	6.41 ± 1.07
DKFZ-PRAD	Illumina HumanHT-12 V3.0 expression beadchip	95	OICR and ICGC	46.97 ± 3.16	30.81 ± 87.31	7.15 ± 0.85
GSE116918	[ADXPCv1a520642] Almac Diagnostics Prostate Disease Specific Array (DSA) (GPL25318)	248	NI Cancer Centre, Belfast Health and Social Care Trust (BHSCT)	67.35 ± 6.36	25.53 ± 26.43	7.48 ± 1.02
MSKCC-PRAD	Affymetrix Human Exon 1.0 ST Array	156	MSKCC	NA	NA	6.84 ± 1.17
SU2C-PRAD	Illumina HiSeq 2500	266	Multiple countries	53.12 ± 21.62	155.22 ± 1048.15	8.02 ± 1.11

OICR, Ontario Institute for Cancer Research; ICGC, International Cancer Genome Consortium. NA, not available.

### Identification of Prognosis-Related Immune Terms and Signature in PRAD

**Figure 3:**
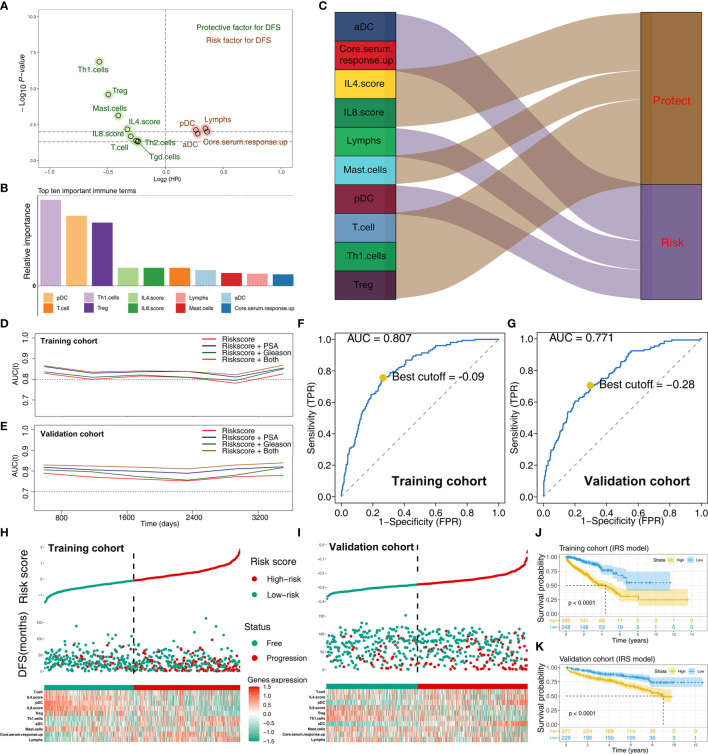
Screening of important immune terms and IRS calculation. **(A)** Volcano plot of 12 prognosis-related immune terms preliminarily identified by univariate Cox analysis with the screening criteria *p*-value <0.05 in training cohort. The red circles represent HR >1 (risk factors), and green circles represent HR <1 (protective factors). **(B)** The top 10 important immune terms based on the relative importance calculated by random forest algorithm and selected for IRS calculation. **(C)** The integrated Sankey diagram illustrated the prognosis effect of these 10 immune terms. **(D, E)** The timeROC plot showed that IRS signature has stable predictive ability of DFS in a different time (training cohort and validation cohort). **(F, G)** The optimum cutoff points for the IRS was established using the ROC analysis (training cohort and validation cohort). **(H, I)** The risk plot showed a higher percentage of progressed patients in the high IRS group (training cohort and validation cohort). **(J, K)** Kaplan-Meier curve of the DFS prognosis in high and low IRS group.

To explore the effect of identified immune terms on patient prognosis, patients with complete survival status were selected for further analysis. In training cohort (TCGA-PRAD, GSE70768, GSE70769), univariate Cox regression analysis showed that 12 immune terms were significantly associated with disease-free survival (DFS) (**Figure 3A** and **Supplementary Table S1**; *p*-value <0.05). The terms were then filtered using random survival forest algorithm based on importance screening. The top 10 important terms, pDC, T cells, Th1 cells, Treg, IL-4 score, IL-8 score, lymphs, mast cells, aDC, and core serum response up were selected for multivariate Cox regression analysis (**Figure 3B** and **Supplementary Figure S1**). The IRS was then calucated using these terms using the formula: IRS = −0.21274 * Th1 cells + 0.35886 * pDC + −0.21017 * Treg + −0.00588 * IL4.score + −0.20957 * T cells + −0.055286 * IL-8 score + 0.21591 * aDC + −0.86202 * mast cells + −0.0502 * lymphs + 0.03938 * core serum response up. Analysis showed that Th1 cells, IL-4 score, Treg, IL-8 score, T cells, and mast cells are protective factors of PRAD prognosis, whereas aDC, pDC, core serum response up, and lymphs were risk factors (**Figure 3C** and **Supplementary Figure S2**). Time AUC curves showed that the IRS for the training cohort performed well in predicting the progression of PRAD patients (AUC >0.8 at the most time) (**Figure 3D**). In the validation cohort, the IRS model also performed well (**Figure 3E**). Moreover, we found that the model combined with IRS and patients’ clinical features (PSA and Gleason) could effectively improve model predicted efficacy (**Figures 3D, E**). Analysis using SimDesign and tdROC package showed that −0.09 and −0.28 are the optimum cutoff values of IRS in the training and validation cohorts (**Figures 3F, G**). PRAD patients were grouped into high and low IRS groups based on the cutoff value, and the results showed that high IRS patients were more inclined to have disease progression (**Figures 3H, I**). Analysis showed that PRAD patients with high IRS were associated with worse DFS prognosis compared with patients with low IRS (**Figures 3J, K**).

### Clinical Correlation and Biological Function of IRS

**Figure 4:**
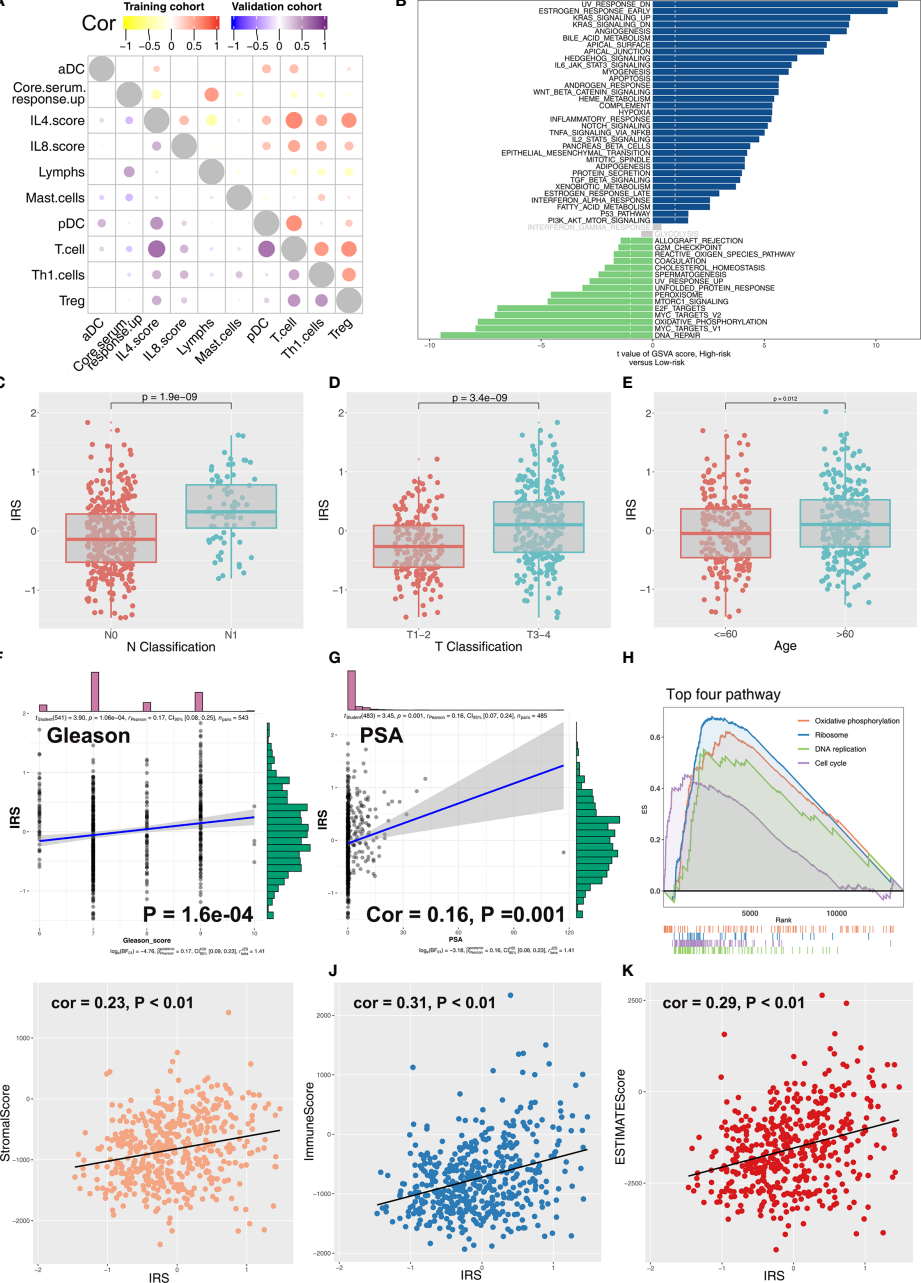
Correlation between IRS and clinical features of PCa patients. **(A)** Coexpression relationship between 10 model immune terms. **(B)** GSVA analysis of IRS signature. The upregulated pathways in high IRS patients are shown in dark blue and the downregulated pathways are shown in green. **(C–G)** The correlation between IRS value and N classification, T classification, age, gleason score, and PSA. **(H)** GSEA analysis of IRS signature. **(I–K)** The correlation between IRS value and immune score, stromal score, and estimate score.

Coexpression relationship of 10 model immune terms was visualized as a correlation coefficient heatmap to further explore the underlying interactions (**Figure 4A**). Furthermore, correlation and biological function analyses were performed to explore the possible mechanism of IRS on patient prognosis. Patients in the high IRS group showed angiogenesis, KRAS signaling, early estrogen response, androgen response, and bile acid metabolism as the most upregulated pathways, whereas E2F target, oxidative phosphorylation, MYC target, and DNA repair were the most downregulated pathways (**Figure 4B**; **Supplementary Table S2** and **Supplementary Figure S3**). In addition, we explored the IRS difference in PRAD patients with different clinical status. Patients with worse pathologic stage showed higher IRS values (N0 vs. N1; T1–2 vs.T3–4) (**Figures 4C, D**). Moreover, older patients showed higher IRS compared with younger patients (**Figure 4E**). Furthermore, IRS showed a positive correlation with Gleason score and the preoperative prostate-specific antigen (PSA) (**Figures 4F, G**). GSEA analysis and KEGG analysis showed that oxidative phosphorylation, DNA replication, cell cycle, and ribosome pathways were upregulated in patients in the high IRS group (**Figure 4H**).

### Correlation of Tumor Microenvironment and Genomic Features With IRS in PRAD

**Figure 5:**
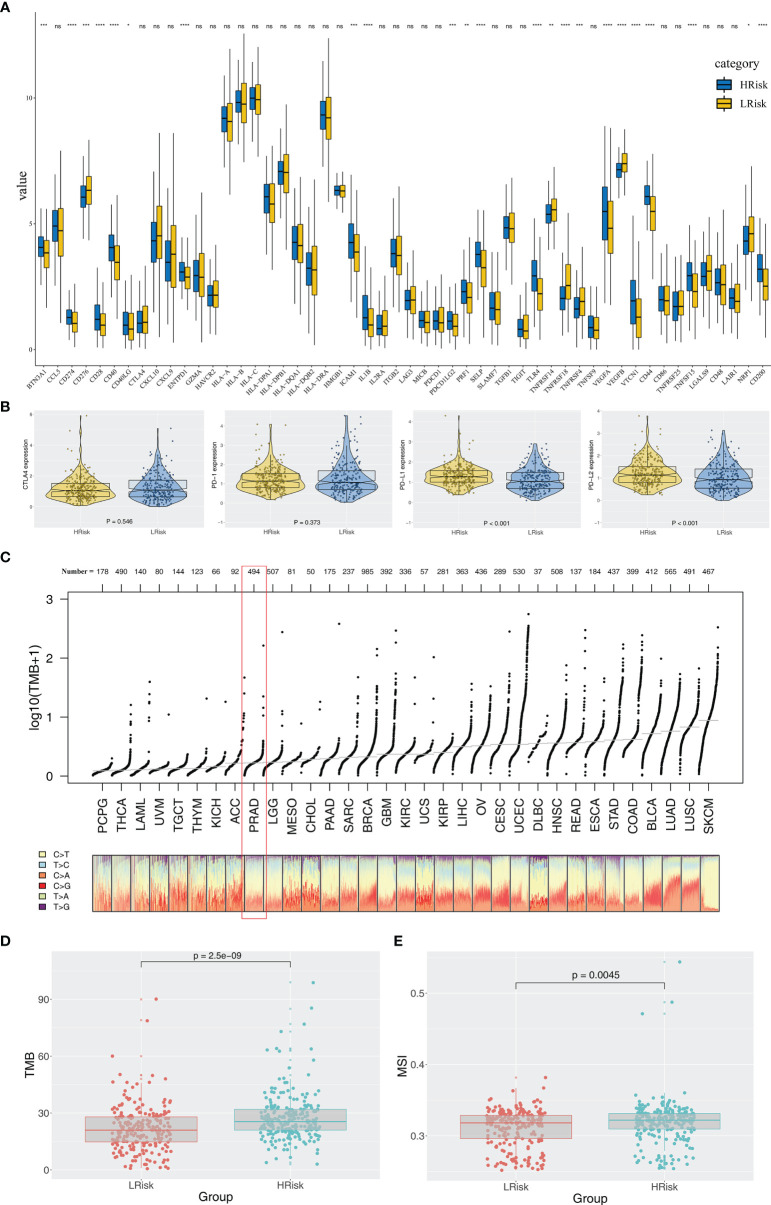
Exploration of the difference of immune checkpoint genes, TMB, and MSI in high and low IRS groups. **(A)** Significant differences were observed in multiple immune checkpoint genes between high and low IRS groups. **(B)** High IRS PRAD patients showed a higher level of PD-1. **(C)** Overview of TMB distribution in TCGA pan cancer. **(D, E)** High IRS PRAD patients showed a higher level of TMB and MSI. *P < 0.05; **P < 0.01; ***P < 0.001; ****P < 0.0001; ns, no significance.

**Figure 6:**
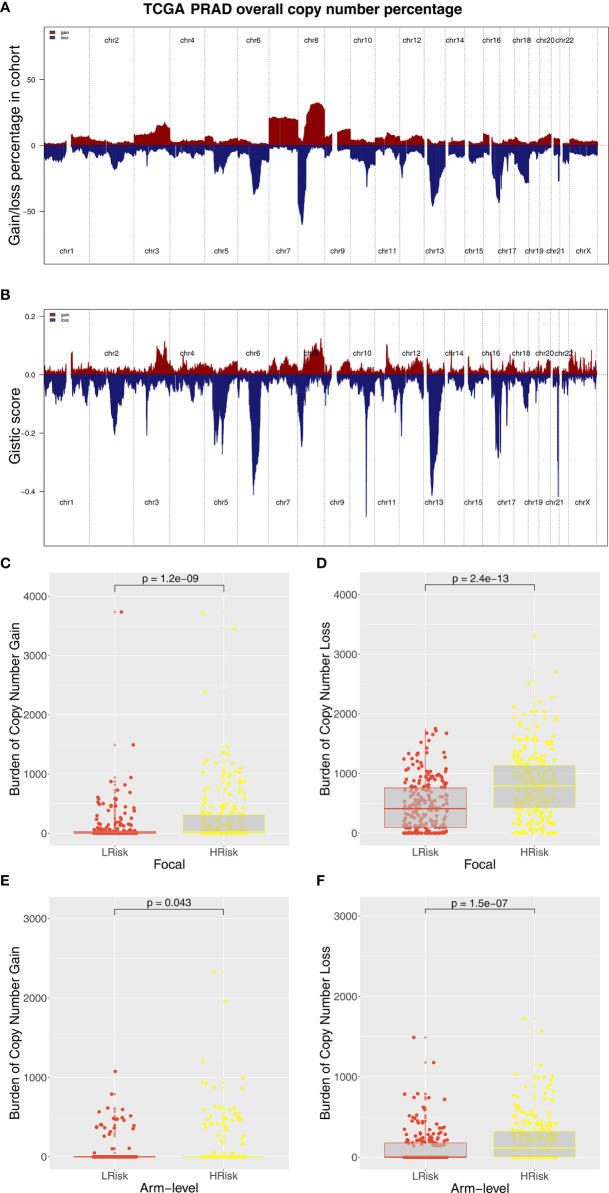
Composite copy number profiles of TCGA-PRAD. **(A)** The gain and loss percentage of copy number profiles of TCGA-PRAD. **(B)** The gistic score of copy number profiles of TCGA-PRAD. **(C, D)** High IRS PRAD patients showed a higher focal level of CNV burden. **(E, F)** High IRS PRAD patients showed a higher arm level of CNV burden.

**Figure 7:**
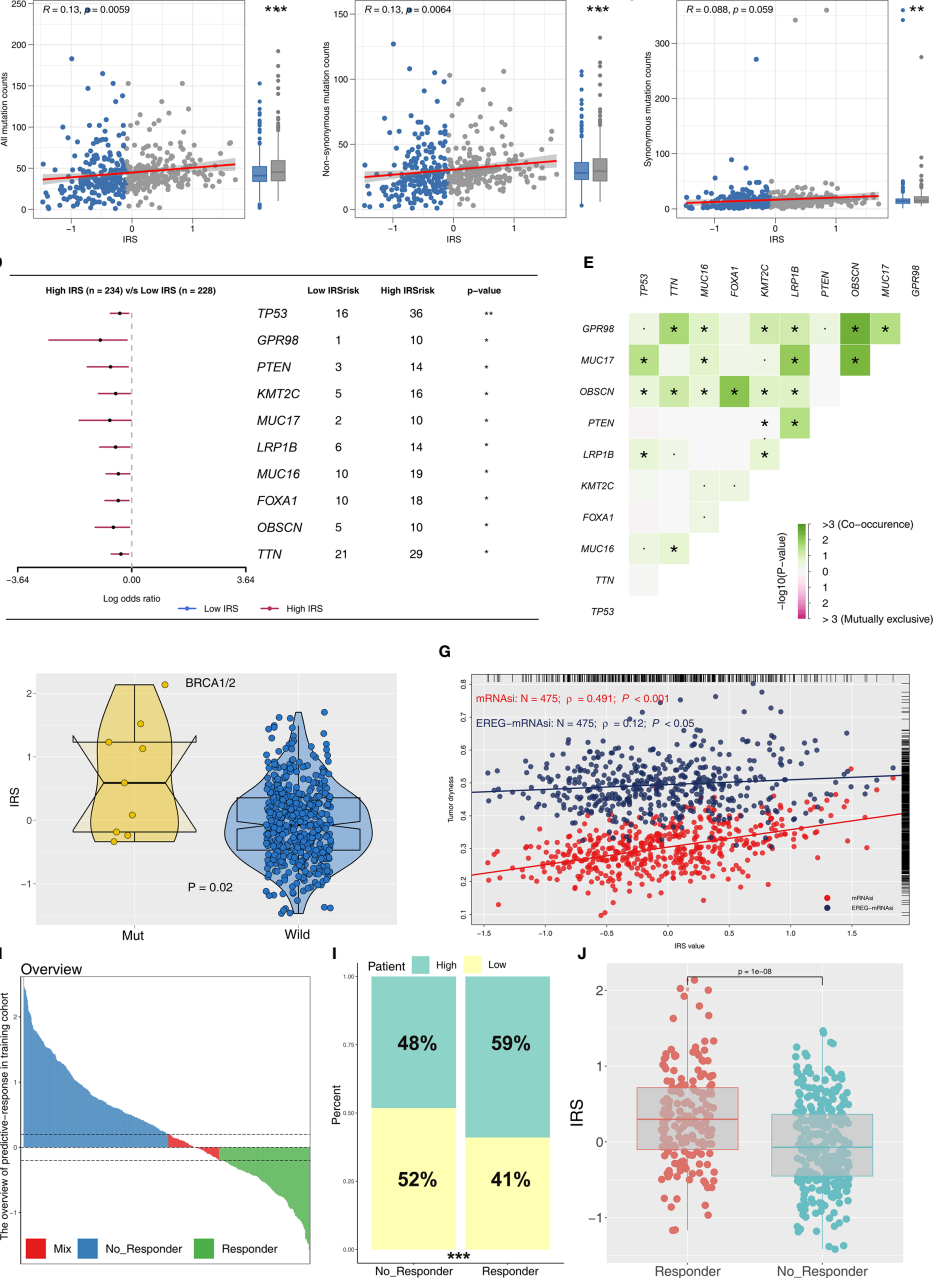
Correlation of IRS with mutation, tumor stemness, and immunotherapy response rate. **(A–C)** The association between genome mutation counts and IRS. **(D)** Gene mutation difference between high and low IRS patients. **(E)** Comutation relationship of mutation genes. **(F)** IRS value in BRCA wild and mutation samples. **(G)** IRS was positively associated with tumor stemness (mRNAsi and EREG-mRNAsi). **(H)** The overview of predictive immunotherapy response rate in the combined PCa cohort using the TIDE analysis. The patients with TIDE value <−0.2 were regarded as responders and those >0.2 were regarded as nonresponders. **(I)** The immunotherapy responders have a higher percentage of high IRS patients. **(J)** The immunotherapy responders have a higher IRS value. *P <0.05; **P < 0.01; ***P < 0.001.

Immune score, stromal score, and estimate score were higher in the PRAD samples of the high IRPS subtype (**Figure 4I–K**). Immune checkpoint modules play important roles in tumor; therefore, we performed correlation analysis between IRS and multiple checkpoint modules. Correlation analysis showed a significant difference in levels of several immune checkpoint modules between high and low IRS groups (**Figure 5A**). High IRS PRAD patients showed a higher level of PD-L1 and PD-L2 (**Figure 5B**). This finding implies that high and low IRS PRAD patients have differences in immunotherapy response. TMB, MSI, and CNV are correlated with immunotherapy response rate for cancer patients. Therefore, we compared TMB, MSI, and CNV differences in the two IRS subtypes. TMB level of all cancer types in the TCGA database were evaluated, and a relatively low level of TMB of PRAD was observed compared with other cancers (**Figure 5C**). Moreover, patients in the high IRS group showed a higher TMB and MSI compared with patients in the low IRS group (**Figures 5D, E**). Moreover, the copy number profile in TCGA-PRAD patients, including gain/loss percentage and gistic score was characterized (**Figures 6A, B**). CNV burden analysis showed higher level of focal and broad CNV burden in patients in high IRS compared with the level in patients in the low IRS group (**Figures 6C–F**). We further explored the association between genome mutation and IRS model. We found a positive correlation between IRS and genome total mutation counts and nonsynonymous mutation counts but not in synonymous mutation (**Figures 7A–C**). Analysis of the transcript mutation status in high and low IRS groups showed that *TP53*, *GPR98*, *PTEN*, *KMT2C*, *LRP1B*, *MUC16*, *FOXA1*, *OBSCN*, *MUC17*, and *TTN* were the more common mutations in high IRS patients compared with low IRS group (**Figure 7D**). Comutation relationship between these genes was then explored for the underlying interaction (**Figure 7E**). Meanwhile, we found that BRCA mutation patients tend to have a higher IRS (**Figure 7F**, *p* < 0.05). Previous studies report association of *TP53* gene with tumor stemness. Therefore, we quantified tumor stemness of TCGA-PRAD to understand the tumor microenvironment difference. Analysis showed a significant positive correlation between IRS and PRAD stemness (mRNAi and EREG-mRNAi; **Figure 7G**).

### IRS Effectively Predicts Immunotherapy Response

**Figure 8:**
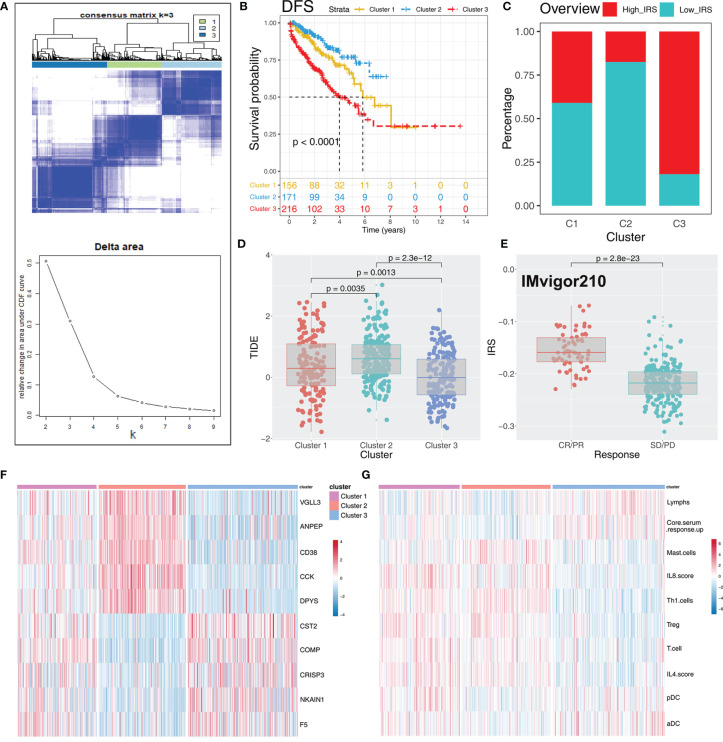
Consensus clustering identified distinct IRS clusters with different DFS prognosis and immunotherapy response rate. **(A)** ConsensusClusterPlus package was used to distinguish IRS subgroups of PRAD with resamplings set as 1,000. The classification was optimized and clustered of samples into three subtypes. **(B)** Kaplan-Meier survival curve showed different prognosis characteristic in IRScluster1–3, among which cluster 2 had the best prognosis and cluster 3 had the worst. **(C)** The IRScluster1–3 had a different percentage of high IRS patients and the IRScluster5 had the most. **(D)** The TIDE difference in IRScluster1–3 and the IRScluster3 had the lowest TIDE value. **(E)** IRS difference in IMvigor210 dataset (CR/PR *vs*. SD/PD). CR, complete response; PR, partial response; SD, stable disease; PD, progressive disease. **(F, G)** The differential gene and immune infiltrating patterns were illustrated in heatmap across five IRSclusters.

To explore the role IRS changes on response of PRAD patients to immunotherapy, we performed TIDE analysis to calculate the TIDE score of each patient. The TIDE score represents the status of immune escape (**Figure 7H**). TIDE analysis showed a higher percentage of high IRS patients in the responder group (**Figure 7I**; 59% vs. 48%; *p* < 0.001). Notably, immunotherapy responder patients showed a higher IRS value compared with no-responder patients (**Figure 7J**). Top 20 differentially expressed genes between high and low IRS patients were used for molecular subtype identification using ConsensusClusterPlus package. The number of iterations was set at 1,000 times to ensure stability of classification categories, and the patients were then classificated into three subtypes (**Figure 8A**). A survival analysis was then conducted to explore prognosis difference in distinct molecular subtypes. Kaplan-Meier survival curves showed that IRScluster2 had the best DFS prognosis, whereas IRScluster3 showed the worst prognosis (**Figure 8B**: training cohort and **Supplementary Figure S4A**: validation cohort). In addition, IRScluster3 showed the highest percentage of high IRS patients, whereas IRScluster2 had the lowest percentage of high IRS patients (**Figure 8C**). Furthermore, IRScluster3 showed a lower TIDE value compared with that of IRScluster1–2, implying IRScluster3 had a higher response rate to immunotherapy (**Figure 8D**: training cohort; **Supplementary Figure S4B**: validation cohort). The results based on the IMvigor210 cohort showed that complete response and partial response (CR/PR) has a significantly higher IRS compared with stable disease and progressive disease (SD/PD) patients (**Figure 8E**). A heatmap showed that *VGLL3*, *ANPEP*, *CD38*, *CCK*, *DPYS*, *CST2*, *COMP*, *CRISP3*, *NKAIN1*, and *F5* genes were differentially expressed in these three clusters (**Figure 8F**). Treg, Th1 cells, IL-4 score, and IL-8 score were the significantly differentially expressed terms in the tumor microenvironment of IRScluster1 to IRScluster3 (**Figure 8G**).

### Candidate Compounds Targeting the IRS Signature Based on CMap Analysis

CMap analysis screens potential compounds and drugs by investigating the changes in genes between different groups, based on a comprehensive and well-curated data resource (26). The top 150 upregulated and downregulated DEGs were identified from IRS high and low groups for CMap analysis. Thioridazine, trifluoperazine, 0175029-0000, trichostatin A, and fluphenazine were potential targets for high IRS PRAD patients (**Table 2**).

**Table 2:** The compound (perturbagen) from the CMap after a range of screening.

CMap name	Cell line	Trial number	Enrichment	*p*-value	Specificity	Percent nonnull	Potential mechanisms of action
Thioridazine	PC-3	5	−0.872	<0.001	0.073	100	Dopamine receptor antagonist
Trifluoperazine	PC-3	3	−0.866	0.033	0.153	100	Dopamine receptor antagonist
0175029-0000	PC-3	4	−0.849	0.049	0.113	100	Cyclin-dependent kinase inhibitor
Trichostatin A	PC-3	55	−0.763	<0.001	0.109	100	Class I/II HDAC inhibitor
Fluphenazine	PC-3	3	−0.846	0.0083	0.089	100	Dopamine receptor antagonist

HDAC, histone deacetylase.

## Discussion

PCa is the most common nonskin cancer, with approximately 1,600,000 new cases and 366,000 cancer mortalities each year worldwide (2, 27). Previous studies have explored immunotherapy with positive results making it an important cancer treatment approach (27). PCa, an indolent tumor, is an ideal therapeutic vaccine model against cancer as it can provide enough time for the occurrence of an antitumor immune response (28). Multiple effective therapy options have significantly improved prognosis of metastatic castration-resistant prostate cancer (mCRPC). Approval of six new drugs in 2010 lays a basis for further development of oncology-immunotherapy. Traditional surgical castration or androgen-deprivation therapy and immunotherapy play key roles in the treatment of PCa. In this study, we developed a new scoring tool named IRS based on 53 immune terms quantified by ssGSEA. The findings of this study show that IRS is significantly correlated with DFS prognosis. In addition, high IRS patients show poor clinical outcomes and genomic features. The IRS accurately predicted the response rate of PCa patients to immunotherapy.

Sva package was used to combine eight different PRAD cohorts into a large population cohort (1,597 samples), thus improving the stability of our analysis and conclusions. In the training cohort, a total of 12 immune terms significantly correlated with patient prognosis were identified using univariate Cox regression analysis based on quantified immune term matrix. Ten immune terms including pDC, T cells, Th1 cells, Treg, IL-4 score, IL-8 score, lymphs, mast cells, aDC, and core serum response up were selected for IRS calculation based on random forest algorithm, indicating their vital role in PCa progression. Th1 cells were the most correlated with PCa progression. Previous studies report that Th1 cells and other immune terms play important roles in cancer progression. For instance, a previous study reports that Th1, Th2, Treg, and Th17 cells in the tumor microenvronment are correlated with prognosis of colorectal cancer (29). In addition, cancer-associated fibroblasts are implicated in robust regulation of extracellular matrix composition, further promoting tumor growth and invasion thus playing an important role in tumor progression (30). Furthermore, Ohue et al. report that Treg is recruited to TME through gradient concentrations of chemokines, contributing to tumorigenesis and development by inhibiting tumor immune status in most cancer types (31). The findings of this study include a positive correlation between Treg and prognosis of PCa patients, indicating a difference between PCa TME and TME of other tumors. With advances in tumor immunotherapy, the key immune terms reported in this study provide a basis for further exploring immune microenvironment of PCa.

Analysis showed that high IRS is associated with poor clinical features, high Gleason score, and PSA, which may be associated with immune inhibition in high IRS patients. Genomic enrichment analysis showed that angiogenesis, Kirsten rat sarcoma virus (KRAS) signaling, early estrogen response, androgen response, and bile acid metabolism were upregulated in high IRS patients. Vasto et al. reported that the early inflammatory responses caused by elevated estrogen result in prostate-specific inflammatory response, promoting the development of PCa (32). Ma et al. analyzed four liver carcinoma mice models and reported that bile acid metabolism promotes recruitment of NKT cells, thus inhibiting cancer growth (33). KRAS and P53 signaling are common pathways in cancers. Yang et al. reported that MAZ induces prostate cancer bone metastasis by transcriptionally activating the KRAS-dependent RalGEF pathway (34). Pascal et al. report that simultaneous inactivation of EAF2 and P53 activates STAT3 and promotes PCa tumorigenesis (35). The findings of the present study showed that high IRS patients might aberrantly activate the above pathways, leading to worse clinical and genomic outcomes.

Moreover, the high IRS group showed a significantly high PD-L1 and PD-L2 level. Levels of PD-1/PD-L1, the main checkpoint of the human immune system, in the tumor microenvironment is associated with the response rate of immunotherapy (36). The immune system easily recognizes and kills tumor cells with high genomic instability, which could affect the response rate of immunotherapy. Liu et al. reported that a combination of TMB and CNV is a better predictor for prognosis and clinical response to immune checkpoint inhibitors (ICI) (37). In addition, Ganesh et al. reviewed multiple ICI-associated clinical trials and reported that high MSI patients have a higher objective remission rate (38). Therefore, genomic instability analysis was used in this study to explore potential correlation between IRS value and immunotherapy response. The level of TMB, MSI, and CNV were significantly higher in high IRS patients. Moreover, *TP53*, *GPR98*, and *PTEN* mutation showed a higher frequency in high IRS patients compared with low IRS patients. Hamid et al. reported that *TP53* and *PTEN* mutation could directly promote PCa progression in PCa samples (39). In a different study by Jamaspishvili et al., cancer-associated *PTEN* mutation results in a strong association with adverse pathological features and oncological outcomes (40). Quantification of tumor stemness showed a positive correlation between IRS and tumor stemness index. Higher tumor stemness index is correlated with poor prognosis and high invasion, which might be the reasons for the poor clinical outcomes in high IRS patients. These findings show that IRS is a good predictor of immunotherapy response rate. Furthermore, we validated these results using TIDE analysis. A higher percentage of responders was observed in the high IRS group compared with the low IRS group. Genotyping of DEGs between high and low IRS patients showed three distinct clusters. Patients in these three clusters showed different prognosis and TIDE values, indicating that they had different response rates to immunotherapy. Furthermore, a regular expression pattern was observed for *VGLL3*, *ANPEP*, *CD38*, *CCK*, *DPYS*, *CST2*, *COMP*, *CRISP3*, *NKAIN1*, and *F5* in the clusters. Identification of these characteristic genes implies that there is no need for further high-throughput sequencing. These genes can be optimized to a practical gene panel for predicting PCa prognosis and immunotherapy response rate.

The occurrence and development of PCa is the result of a variety of factors. In the tumor microenvironment, the interaction between tumor-infiltrating lymphocytes (TILs) and tumor cells significantly affected the biological behavior of cancer. A recent study has reported that the germline genome variants in NK cells could influence TIL recruitment, immunotherapy response, and clinical outcomes (41). Germline genome may substantially affect immune capacity in cancer patients at a population level, including the response rate to cancer immunotherapy (42). Xue et al. comprehensively analyzed the germline genomes of nearly 20,000 patients in 22 common cancer types and concluded that germline genomic patterns could inform treatment and clinical outcomes (43). Based on the prognosis-related immune terms, we identified IRS model to predict PCa patient survival and immunotherapy response rate. We found that high IRS patients tend to have elevated genome instability, partly explaining its worse prognosis and high immunotherapy response rate.

CMap analysis was used to identify potential compounds and drugs targeting high IRS patients. Thioridazine, trifluoperazine, 0175029-0000, trichostatin A, and fluphenazine were potential targets for high IRS patients, and all the compounds inhibited PCa progression. Some of these compounds have been reported to suppress PCa growth in previous studies. Singh et al. reported that thioridazine inhibits outgrowth of androgen-independent PCa through TLK1/NEK1/DDR axis through *in vitro* and *in vivo* experiments (44). Batra et al. reported that trifluoperazine inhibits PCa cell proliferation by regulating a calmodulin-mediated pathway (45). The findings of this study imply that these compounds limit PCa progression and affect the response rate of immunotherapy. However, further studies should be carried out to further explore the mechanisms of these compounds.

This study had a few limitations which should be addressed in the future. The cohort included in our analysis comprised mainly of Western samples with a few Asian representatives. This bias may affect application of the findings of this study in the Asian population. Therefore, the findings of this study may not represent all PCa patients or may be influenced by diverse genetic backgrounds. Although most of our samples had complete prognosis information (survival time and status), detailed clinical features of each sample, such as TNM classification, chemotherapy, and lifestyle were not included in the open-access data source. However, IRS value was a robust signature for predicting DFS prognosis of PCa patients and was validated in the training and validation cohorts with large populations. Moreover, evaluation of biological mechanism of high IRS patients through a series of bioinformatics analysis showed potential compounds targeting the IRS signature (thioridazine, trifluoperazine, 0175029-0000, trichostatin A, and fluphenazine).

## Conclusion

In summary, our study evaluated the immune environment of PCa patients based on a combined cohort of a large population. The IRS signature based on 10 immune terms was a powerful tool for predicting prognosis and immunotherapy response rate of PCa patients. Furthermore, patients in high IRS group showed poor clinical features, genomic character and biological pathway associated with poor prognosis of PCa. In addition, potential compounds targeting the IRS signature were identified. *VGLL3*, *ANPEP*, *CD38*, *CCK*, *DPYS*, *CST2*, *COMP*, *CRISP3*, *NKAIN1*, and *F5* were identified as characteristic genes implicated in differences in prognosis and immunotherapy response rates in the three IRSclusters.

## Data Availability Statement

The original contributions presented in the study are included in the article/**Supplementary Material**. Further inquiries can be directed to the corresponding authors.

## Author Contributions

XR and CX collected the data and performed all analysis. XR and XZ wrote the manuscript. All the authors participated in the data analysis and approved the final version of the manuscript.

## Funding

This work was supported by the National Natural Science Foundation of China (grant number 81972386).

## Conflict of Interest

The authors declare that the research was conducted in the absence of any commercial or financial relationships that could be construed as a potential conflict of interest.

## Publisher’s Note

All claims expressed in this article are solely those of the authors and do not necessarily represent those of their affiliated organizations, or those of the publisher, the editors and the reviewers. Any product that may be evaluated in this article, or claim that may be made by its manufacturer, is not guaranteed or endorsed by the publisher.

## Supplementary Material

Supplementary Figure 1 The error rate and importance derived from random forest analysis. **(A)** The error rate derived from random forest algorithm with the ntree = 1,000. **(B)** The relative importance of the genes derived from random forest algorithm.

Supplementary Figure 2 Kaplan-Meier survival curve of the top 10 immune terms.

Supplementary Figure 3 Enrichment analysis of IRS signature with all the GSEA gene set.

Supplementary Figure 4 Three cluster identified in training cohort was validated in the validation cohort. Notes: **(A)** Kaplan-Meier survival curves showed that IRScluster2 had the best DFS prognosis, whereas IRScluster3 showed the worst prognosis. **(B)** IRScluster3 showed a lower TIDE value compared with that of IRScluster1–2.
