# Supplementary figures and images for: Immune Microenvironment and Response in Prostate Cancer Using Large Population Cohorts
Source: Front Immunol. 2021 Oct 28;12:686809. doi: 10.3389/fimmu.2021.686809 (PMC8585452; doi:10.3389/fimmu.2021.686809)

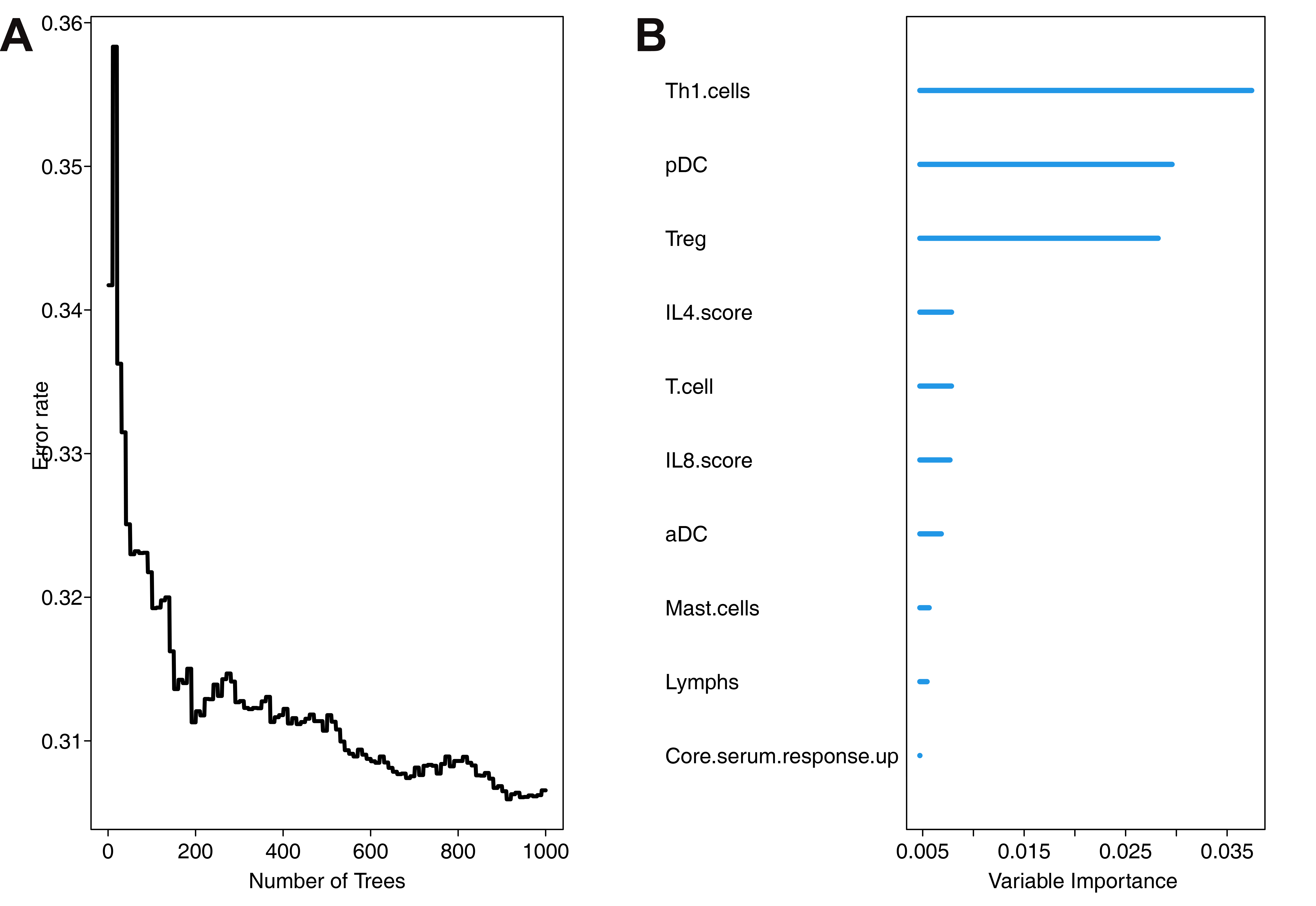

Supplement: Supplementary Figure 1 — The error rate and importance derived from random forest analysis. (A) The error rate derived from random forest algorithm with the ntree = 1,000. (B) The relative importance of the genes derived from random forest algorithm. [file Image_1.tif]

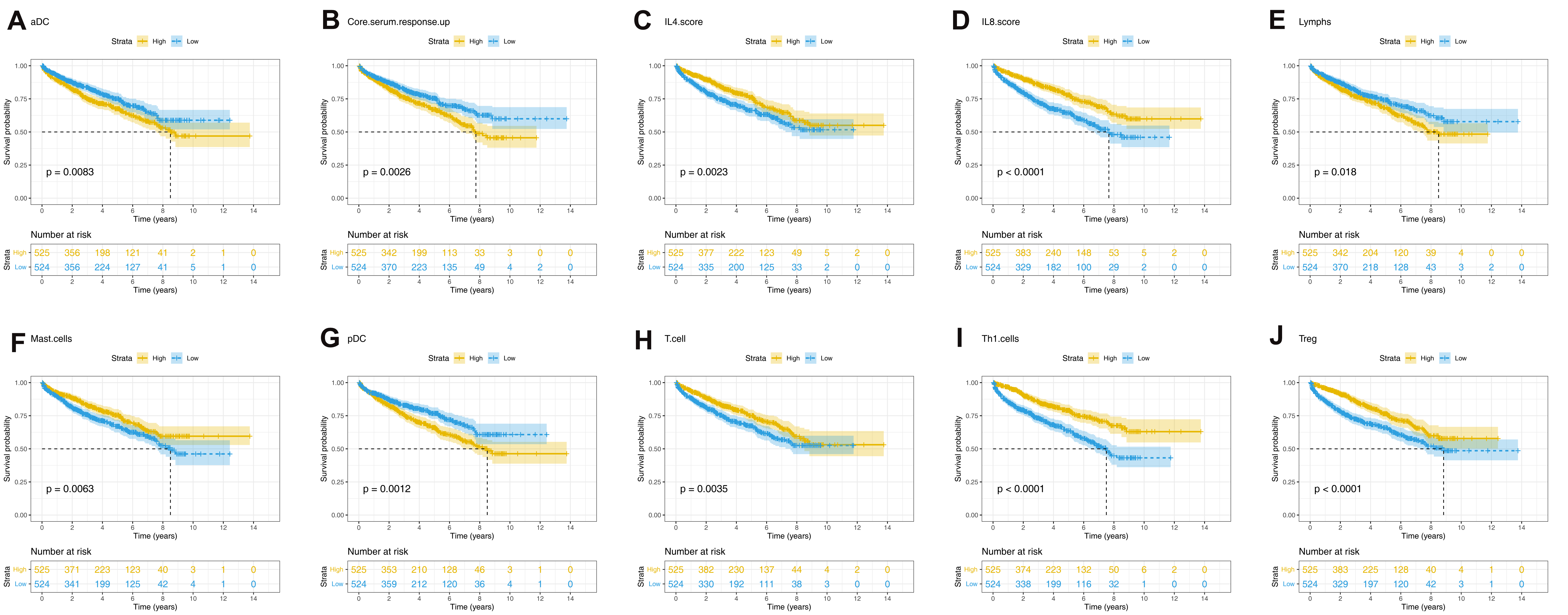

Supplement: Supplementary Figure 2 — Kaplan-Meier survival curve of the top 10 immune terms. [file Image_2.tif]

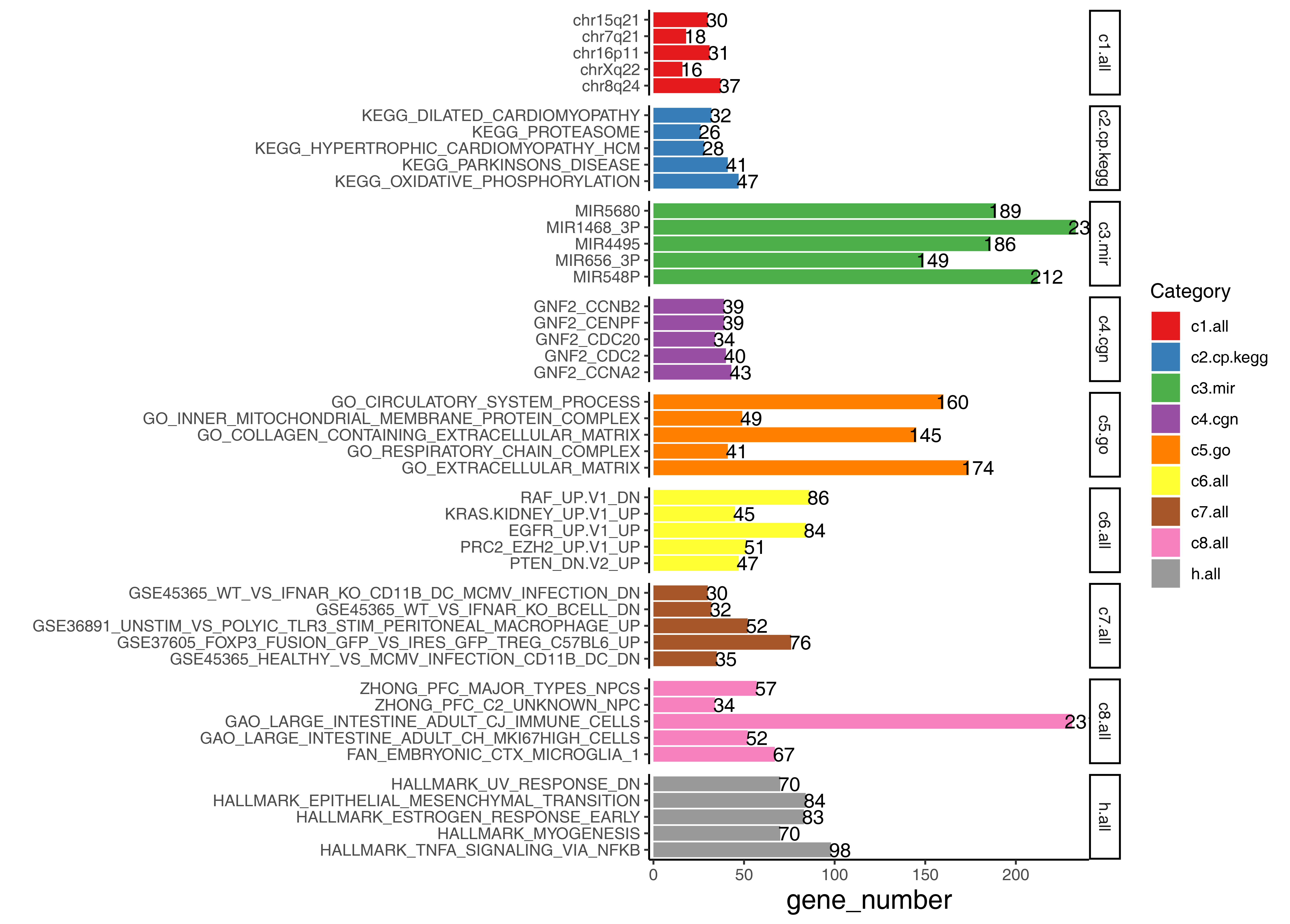

Supplement: Supplementary Figure 3 — Enrichment analysis of IRS signature with all the GSEA gene set. [file Image_3.tif]

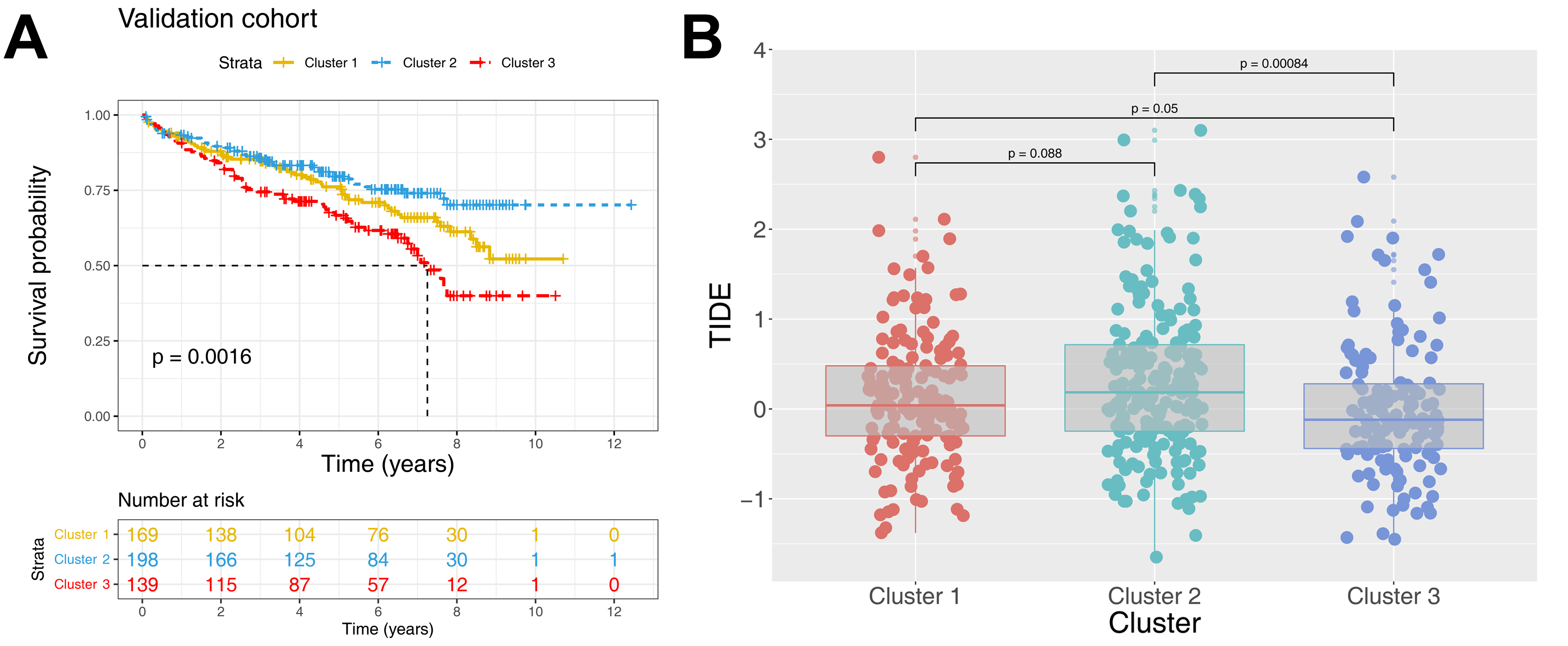

Supplement: Supplementary Figure 4 — Three cluster identified in training cohort was validated in the validation cohort. Notes: (A) Kaplan-Meier survival curves showed that IRScluster2 had the best DFS prognosis, whereas IRScluster3 showed the worst prognosis. (B) IRScluster3 showed a lower TIDE value compared with that of IRScluster1–2. [file Image_4.tif]
