# Supplementary material for: Immune Microenvironment and Response in Prostate Cancer Using Large Population Cohorts
Source: Front Immunol. 2021 Oct 28;12:686809. doi: 10.3389/fimmu.2021.686809 (PMC8585452; doi:10.3389/fimmu.2021.686809)
Supplement: Supplementary file 5 [file Table_1.docx]

| **Table S1. The immune terms associated with the prognosis of PCa patients** | | | | |
| --- | --- | --- | --- | --- |
| Immune term | HR | P.value | Lower 95% Cl | Upper 95% Cl |
| Th1.cells | 0.673 | 0.000 | 0.581 | 0.780 |
| Treg | 0.710 | 0.000 | 0.606 | 0.833 |
| Mast.cells | 0.754 | 0.001 | 0.639 | 0.888 |
| Lymphs | 1.271 | 0.006 | 1.072 | 1.507 |
| IL4.score | 0.796 | 0.007 | 0.675 | 0.939 |
| pDC | 1.200 | 0.008 | 1.050 | 1.373 |
| Core.serum.response.up | 1.284 | 0.010 | 1.062 | 1.553 |
| aDC | 1.213 | 0.014 | 1.040 | 1.415 |
| IL8.score | 0.812 | 0.021 | 0.681 | 0.969 |
| T.cell | 0.839 | 0.043 | 0.708 | 0.994 |
| Th2.cells | 0.849 | 0.046 | 0.717 | 0.994 |
| Tgd.cells | 0.847 | 0.049 | 0.713 | 0.997 |
