# Supplementary material for: Immune Microenvironment and Response in Prostate Cancer Using Large Population Cohorts
Source: Front Immunol. 2021 Oct 28;12:686809. doi: 10.3389/fimmu.2021.686809 (PMC8585452; doi:10.3389/fimmu.2021.686809)
Supplement: Supplementary file 6 [file Table_2.docx]

| **Table S2. All GSVA pathways between high and low IRS patients.** | | | | | | |
| --- | --- | --- | --- | --- | --- | --- |
| **Terms** | **logFC** | **AveExpr** | **t** | **P.Value** | **adj.P.Val** | **B** |
| HALLMARK_UV_RESPONSE_DN | 0.29 | 0.01 | 10.99 | 0.00 | 0.00 | 46.44 |
| HALLMARK_ESTROGEN_RESPONSE_EARLY | 0.18 | 0.01 | 10.52 | 0.00 | 0.00 | 42.39 |
| HALLMARK_DNA_REPAIR | -0.18 | -0.02 | -9.51 | 0.00 | 0.00 | 33.98 |
| HALLMARK_KRAS_SIGNALING_UP | 0.18 | 0.02 | 8.85 | 0.00 | 0.00 | 28.85 |
| HALLMARK_KRAS_SIGNALING_DN | 0.15 | 0.03 | 8.80 | 0.00 | 0.00 | 28.44 |
| HALLMARK_ANGIOGENESIS | 0.36 | 0.02 | 8.69 | 0.00 | 0.00 | 27.68 |
| HALLMARK_BILE_ACID_METABOLISM | 0.15 | 0.01 | 7.94 | 0.00 | 0.00 | 22.22 |
| HALLMARK_MYC_TARGETS_V1 | -0.24 | -0.03 | -7.94 | 0.00 | 0.00 | 22.21 |
| HALLMARK_OXIDATIVE_PHOSPHORYLATION | -0.23 | -0.03 | -7.84 | 0.00 | 0.00 | 21.52 |
| HALLMARK_APICAL_SURFACE | 0.19 | 0.02 | 7.80 | 0.00 | 0.00 | 21.20 |
| HALLMARK_APICAL_JUNCTION | 0.19 | 0.01 | 7.67 | 0.00 | 0.00 | 20.35 |
| HALLMARK_MYC_TARGETS_V2 | -0.23 | -0.04 | -7.08 | 0.00 | 0.00 | 16.44 |
| HALLMARK_E2F_TARGETS | -0.20 | -0.03 | -6.97 | 0.00 | 0.00 | 15.70 |
| HALLMARK_HEDGEHOG_SIGNALING | 0.18 | 0.03 | 6.48 | 0.00 | 0.00 | 12.69 |
| HALLMARK_IL6_JAK_STAT3_SIGNALING | 0.19 | 0.02 | 6.22 | 0.00 | 0.00 | 11.20 |
| HALLMARK_MYOGENESIS | 0.19 | 0.01 | 6.09 | 0.00 | 0.00 | 10.45 |
| HALLMARK_APOPTOSIS | 0.11 | 0.00 | 5.66 | 0.00 | 0.00 | 8.09 |
| HALLMARK_ANDROGEN_RESPONSE | 0.15 | 0.00 | 5.64 | 0.00 | 0.00 | 8.01 |
| HALLMARK_WNT_BETA_CATENIN_SIGNALING | 0.13 | 0.02 | 5.64 | 0.00 | 0.00 | 8.00 |
| HALLMARK_HEME_METABOLISM | 0.10 | -0.01 | 5.44 | 0.00 | 0.00 | 6.96 |
| HALLMARK_COMPLEMENT | 0.12 | 0.01 | 5.36 | 0.00 | 0.00 | 6.56 |
| HALLMARK_HYPOXIA | 0.11 | 0.01 | 5.35 | 0.00 | 0.00 | 6.51 |
| HALLMARK_INFLAMMATORY_RESPONSE | 0.16 | 0.03 | 5.32 | 0.00 | 0.00 | 6.35 |
| HALLMARK_NOTCH_SIGNALING | 0.13 | 0.00 | 5.16 | 0.00 | 0.00 | 5.53 |
| HALLMARK_TNFA_SIGNALING_VIA_NFKB | 0.14 | 0.00 | 5.01 | 0.00 | 0.00 | 4.85 |
| HALLMARK_IL2_STAT5_SIGNALING | 0.10 | 0.00 | 4.77 | 0.00 | 0.00 | 3.72 |
| HALLMARK_MTORC1_SIGNALING | -0.12 | -0.01 | -4.71 | 0.00 | 0.00 | 3.44 |
| HALLMARK_PEROXISOME | -0.09 | 0.00 | -4.56 | 0.00 | 0.00 | 2.79 |
| HALLMARK_PANCREAS_BETA_CELLS | 0.13 | 0.04 | 4.37 | 0.00 | 0.00 | 1.96 |
| HALLMARK_EPITHELIAL_MESENCHYMAL_TRANSITION | 0.14 | 0.02 | 4.23 | 0.00 | 0.00 | 1.39 |
| HALLMARK_MITOTIC_SPINDLE | 0.10 | 0.00 | 4.13 | 0.00 | 0.00 | 0.97 |
| HALLMARK_ADIPOGENESIS | 0.08 | -0.01 | 4.12 | 0.00 | 0.00 | 0.96 |
| HALLMARK_PROTEIN_SECRETION | 0.13 | -0.01 | 4.00 | 0.00 | 0.00 | 0.46 |
| HALLMARK_TGF_BETA_SIGNALING | 0.11 | 0.00 | 3.92 | 0.00 | 0.00 | 0.15 |
| HALLMARK_XENOBIOTIC_METABOLISM | 0.07 | 0.01 | 3.73 | 0.00 | 0.00 | -0.55 |
| HALLMARK_UNFOLDED_PROTEIN_RESPONSE | -0.07 | -0.02 | -3.15 | 0.00 | 0.00 | -2.48 |
| HALLMARK_ESTROGEN_RESPONSE_LATE | 0.06 | 0.02 | 2.98 | 0.00 | 0.00 | -2.98 |
| HALLMARK_UV_RESPONSE_UP | -0.05 | -0.01 | -2.83 | 0.00 | 0.01 | -3.42 |
| HALLMARK_INTERFERON_ALPHA_RESPONSE | 0.08 | 0.01 | 2.57 | 0.01 | 0.01 | -4.12 |
| HALLMARK_FATTY_ACID_METABOLISM | 0.05 | 0.00 | 2.56 | 0.01 | 0.01 | -4.13 |
| HALLMARK_SPERMATOGENESIS | -0.04 | -0.01 | -2.43 | 0.02 | 0.02 | -4.46 |
| HALLMARK_CHOLESTEROL_HOMEOSTASIS | -0.05 | -0.01 | -2.15 | 0.03 | 0.04 | -5.09 |
| HALLMARK_COAGULATION | -0.04 | 0.00 | -1.75 | 0.08 | 0.09 | -5.86 |
| HALLMARK_REACTIVE_OXIGEN_SPECIES_PATHWAY | -0.04 | -0.02 | -1.75 | 0.08 | 0.09 | -5.87 |
| HALLMARK_P53_PATHWAY | 0.02 | -0.01 | 1.60 | 0.11 | 0.12 | -6.12 |
| HALLMARK_PI3K_AKT_MTOR_SIGNALING | 0.04 | 0.00 | 1.58 | 0.11 | 0.12 | -6.13 |
| HALLMARK_G2M_CHECKPOINT | -0.04 | -0.02 | -1.54 | 0.12 | 0.13 | -6.21 |
| HALLMARK_ALLOGRAFT_REJECTION | -0.04 | 0.00 | -1.44 | 0.15 | 0.16 | -6.35 |
| HALLMARK_GLYCOLYSIS | -0.01 | -0.01 | -0.52 | 0.61 | 0.62 | -7.25 |
| HALLMARK_INTERFERON_GAMMA_RESPONSE | 0.01 | 0.01 | 0.39 | 0.70 | 0.70 | -7.31 |
